# Risk factors for first-trimester spontaneous abortion and the role of preconception care

**DOI:** 10.3389/fgwh.2025.1615983

**Published:** 2025-09-23

**Authors:** Yuliya Podilyakina, Leila Stabayeva, Dusentay Kulov, Yevgeniy Kamyshanskiy, Zhanna Amirbekova, Rasa Stundžienė, Olzhas Zhamantayev

**Affiliations:** 1 School of Residency and Professional Development, Karaganda Medical University, Karaganda, Kazakhstan; 2 Department of Morphology, Karaganda Medical University, Karaganda, Kazakhstan; 3 School of Public Health, Karaganda Medical University, Karaganda, Kazakhstan; 4 Clinic of the Karaganda Medical University, Karaganda, Kazakhstan; 5 Department of Obstetrics and Gynecology, Karaganda Medical University, Karaganda, Kazakhstan; 6 Faculty of Medicine, Institute of Health Sciences, Department of Nursing, Vilnius University, Vilnius, Lithuania

**Keywords:** women's health, reproductive health, preconception care, spontaneous abortion, miscarriage, maternal health

## Abstract

**Background:**

Spontaneous abortion in the first trimester is a common adverse pregnancy outcome with significant implications for maternal health and public health practice. The description of associations with modifiable factors, including preconception care, can aid in planning strategies to improve pregnancy outcomes.

**Methods:**

A retrospective analysis was conducted using data from 1,526 women, divided into two groups based on pregnancy outcomes: spontaneous abortion in the first trimester and live births. Binary and multivariate logistic regression analyses were performed to identify associations between factors (including preconception care) and the risk of spontaneous abortion in the first trimester.

**Results:**

Age >35 years [[OR] = 2.02, 95% [CI] = 1.49–2.75], obesity [[OR] = 1.81, 95% [CI] = 1.12–2.91], and a history of spontaneous abortion [[OR] = 1.57, 95% [CI] = 1.01–2.43] were associated with higher odds of spontaneous abortion in the first trimester, whereas preconception care was associated with lower odds of spontaneous abortion in the first trimester [[OR] = 0.58, 95% [CI] = 0.45–0.75].

**Conclusion:**

The findings may help clinicians stratify pregnant women who require additional monitoring and pre-pregnancy interventions. From a public health perspective, integrating preconception care into routine health services can enhance maternal and neonatal outcomes, reduce healthcare costs, and improve health equity by targeting vulnerable populations. However, the results should be interpreted as associations, and prospective studies are needed to assess the potential effects of preconception care on spontaneous abortion in the first trimester.

## 1 Introduction

Spontaneous abortion (SA), classified under ICD-10 code O03 and commonly referred to as miscarriage, is a prevalent complication in early pregnancy, with incidence rates varying depending on population characteristics and study methodologies (1, 2). Research indicates that approximately 10% to 20% of clinically recognized pregnancies worldwide result in SA, with higher rates observed when early pregnancy losses are included (3, 4). The majority of these losses occur in the first trimester (5, 6). In Kazakhstan, a study involving 237 participants reported that 25.6% of respondents had experienced SA (7). A large-scale national study analyzing 207,317 records of women across all regions of Kazakhstan estimated the prevalence of spontaneous pregnancy loss before 22 weeks of gestation to be 8.7%, with a 20% decline observed over the 2014–2019 period (8).

Several factors have been associated with an increased risk of SA. Advanced maternal age is a significant risk factor, as chromosomal abnormalities in embryos are more common in older women (9, 10). A cross-sectional study in Astana, Kazakhstan (2015–2017), analyzing 67,759 inpatient records, found advancing maternal age significantly increased the risk of miscarriage, with women aged 30–39 and 40+ years having 54% and 272% higher risks, respectively compared to women aged 19–29 years (11). Moreover, maternal obesity has been linked to higher miscarriage rates, potentially due to hormonal imbalances and metabolic disturbances, with risks ranging from 1.25 to 2.25 times higher compared to normal-weight women (12–14). Another important factor in early pregnancy loss is the history of SA and pre-pregnancy health. Studies have shown that a history of SA significantly increases the risk of subsequent losses, with women who have experienced one or more miscarriages being at higher risk for recurrence (15, 16). Inadequate pre-pregnancy health, including suboptimal management of chronic conditions, poor nutritional status, and lack of preconception care, has been linked to adverse pregnancy outcomes (17). Lifestyle factors prior pregnancy, including smoking and alcohol consumption, have also been implicated in elevating miscarriage risk (18, 19).

Building on these findings, it becomes clear that preconception may play an important role in addressing the modifiable risk factors associated with early pregnancy loss (20). Preconception care, as defined by the World Health Organization, includes a set of biomedical, behavioral, and social interventions provided to women and couples before conception to improve maternal and fetal outcomes (21). It aims to identify and mitigate risk factors such as obesity, unmanaged chronic conditions, and inadequate nutritional status while promoting healthier lifestyle choices (22). The benefits of preconception care are well-documented, with evidence indicating that it can help reducing the incidence of adverse pregnancy outcomes, including SA, preterm birth, and congenital anomalies (23). Targeted preconception interventions, such as weight management, smoking cessation, and folic acid supplementation, have been shown to improve pregnancy outcomes and reduce healthcare costs by preventing complications before conception (24).

This study aimed to investigate the socio-demographic and clinical risk factors associated with SA in the first trimester and evaluate the protective effects of preconception care among women in Kazakhstan, largest country in Central Asia. It adds a global health perspective and highlights the importance of addressing disparities in maternal health outcomes, an essential aspect of public health research. While previous studies have extensively documented risk factors for spontaneous abortion, there is limited research on the role of preconception care in mitigating these risks, particularly in low- and middle-income countries (LMICs), and some finding can be extrapolated to all Central Asian countries.

## 2 Materials and methods

### 2.1 Study design

This retrospective study included women who received health services for SA in the first trimester or during childbirth at the State Clinical Hospital, a large conglomerate that includes two perinatal centers of the first and second levels in Karaganda, Kazakhstan, from January 1, 2018, to January 1, 2023. Women from across the country, including those with complicated medical histories, are admitted to these perinatal centers, as they provide the most advanced medical care. The first-level perinatal center offers basic obstetric and neonatal care for low-risk pregnancies and routine deliveries, while the second-level perinatal center provides specialized care for moderate and high-risk pregnancies and newborns requiring additional medical attention.

The use of clinical data from women and morphological data of SA tissue for research purposes was approved by the Local Commission on Bioethics of Karaganda Medical University. Written informed consent was obtained from all pregnant women for the use of their clinical and morphological data in the study.

**Figure 1:**
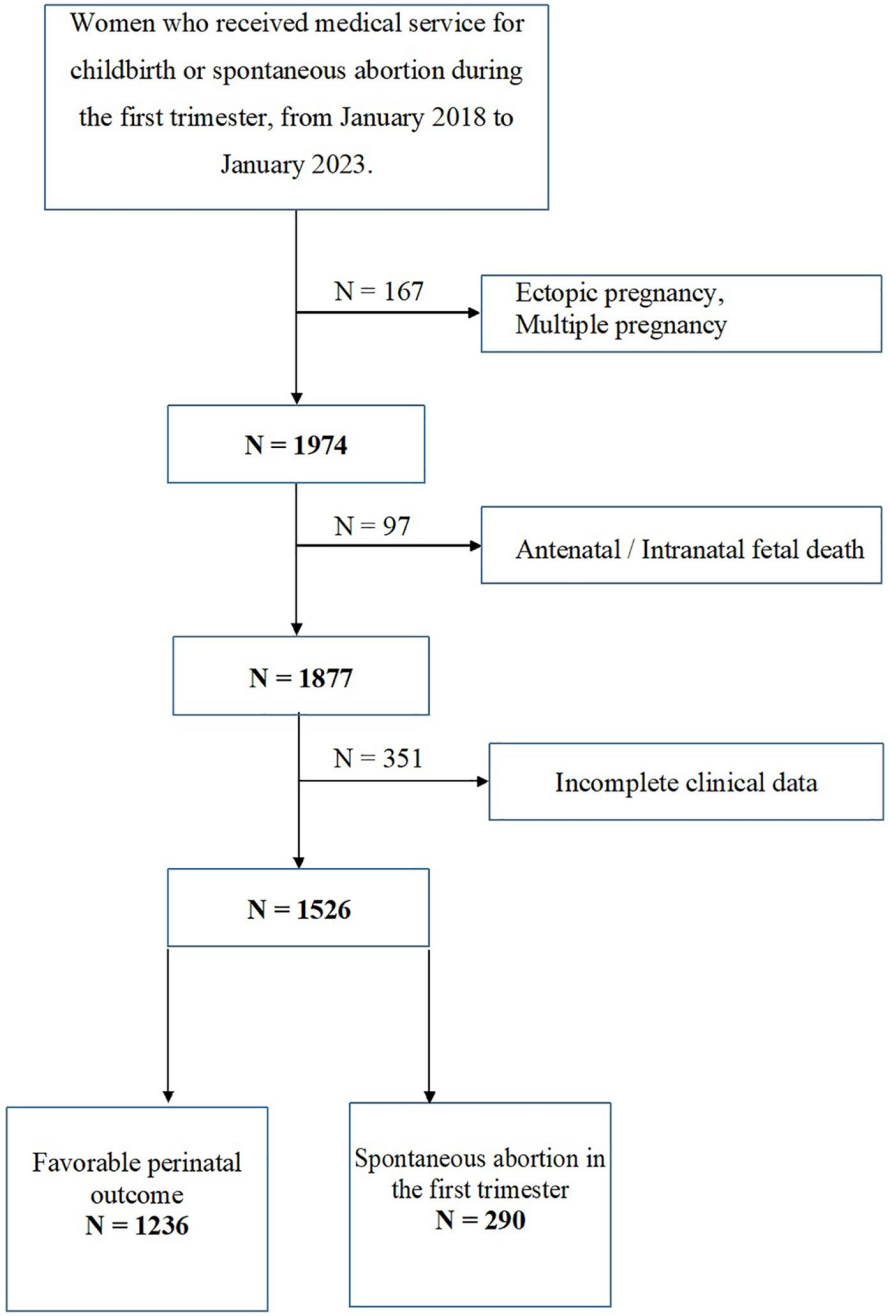
Flowchart illustrating the recruitment process of study participants.

Women's clinical and sociodemographic data were obtained from the Integrated Health Information System (IHIS) and reviewed for missing values. Patients with incomplete data were excluded from the final analysis (Supplementary Table S1). Women with ectopic pregnancies, multiple pregnancies, or antenatal or intrapartum fetal deaths were also excluded. The study block diagram is presented in Figure 1.

The study included data from 1,526 women, divided into two groups:

1. Favorable outcome (*n* = 1,236): Pregnancy resulting in live birth.
2. SA in the first trimester (*n* = 290): Pregnancy ending in spontaneous abortion.

Variables extracted from IHIS included women's age at the time of the study, ethnicity, place of residence, social status, education, marital status, body mass index (BMI), type 1 or type 2 diabetes, arterial hypertension, hypothyroidism, deficiency anemia, allergies, health insurance status before pregnancy, parity (divided into two groups), and preconception care. Preconception care interventions included nutritional counseling, folic acid supplementation, management of chronic conditions (e.g., diabetes, hypertension), and lifestyle modifications (e.g., weight management). These services were provided to women who sought care at the State Clinical Hospital in Karaganda.

The primary outcome variable was the pregnancy outcome, classified into two groups: “First-trimester SA” and “Favorable outcome.” A favorable outcome was defined as the birth of a live newborn. The diagnosis of first-trimester SA was made clinically, based on bleeding and apparent expulsion of the embryo or fetus (confirmed by histological examination), or through ultrasound.

The first trimester was chosen as the cut-off point because SA occurring before this period typically shares a common pathophysiological etiology, such as inflammation, luteal phase deficiency, or chromosomal and structural abnormalities, which differ from miscarriages occurring after the first trimester.

The date of birth of each study participant was used to calculate their age. Age was classified into three categories: 18–26 years, 27–35 years, and over 35 years. Minors were excluded from the study.

### 2.2 Definitions

First-trimester SA: Spontaneous abortion occurring before the 13th week of gestation (25, 26).

Favorable outcome: Defined in this study as a pregnancy resulting in live birth.

In this study, preconception care referred to a set of measures conducted six or more months before pregnancy and documented in patients’ medical records. These included nutritional counseling (with recommendations for folic acid intake), screening and management of chronic diseases as indicated, medical-genetic counseling when necessary, testing for HIV infection, hepatitis B and C, and sexually transmitted infections, as well as recommendations for lifestyle modifications for both spouses (body weight control, physical exercise, and cessation of harmful habits). These medical services were provided in accordance with the recently updated standard of obstetric and gynecological care in the Republic of Kazakhstan (27).

BMI before pregnancy: Calculated as the registered weight (kg) divided by the square of measured height (m) and categorized into four groups. For Caucasians: underweight (<18.5 kg/m^2^), normal weight (≥18.5 and <25 kg/m^2^), overweight (≥25 and <30 kg/m^2^), and obese (≥30 kg/m^2^). For Asians: underweight (<18.5 kg/m^2^), normal weight (≥18.5 and <22.9 kg/m^2^), overweight (≥23 and <24.9 kg/m^2^), and obese (≥25 kg/m^2^) (28, 29).

Diabetes mellitus was diagnosed based on HbA1c ≥ 6.5% (48 mmol/mol) and/or fasting plasma glucose or 2 h post-oral glucose tolerance test glucose levels ≥7.0 mmol/L.

Hypertension: Defined as a systolic blood pressure ≥140 mmHg or a diastolic blood pressure ≥90 mmHg in two separate measurements taken before 20 weeks of gestation (30).

Hypothyroidism was identified based on a pre-pregnancy diagnosis with thyroid-stimulating hormone (TSH) levels ≥ 2.5 mIU/L or, in the first trimester, TSH > 4 mIU/L.

Anemia: Defined as hemoglobin (Hb) < 110 g/L and hematocrit <33%, or as a transferrin saturation (TSat) <16%, ferritin concentration <30 µg/L, or vitamin B12 concentration <200 pg/ml (31).

### 2.3 Statistical analysis

Statistical analysis was performed using IBM SPSS Statistics (version 25.0, IBM Corp., Armonk, NY, USA). Percentages and frequencies were calculated for categorical variables, while the normality of continuous variables was tested using the Kolmogorov–Smirnov test. The Mann–Whitney *U* test was used to examine group differences for continuous variables. For categorical variables, comparisons were made using the chi-square test.

Associations between potential factors (including preconception care) and first-trimester spontaneous abortion were assessed using univariable and multivariable logistic regression. The outcome was coded as SA = 1 (adverse outcome). Discrimination of the final adjusted multivariable model was evaluated using the area under the receiver operating characteristic curve (AUC), calculated based on the predicted probabilities of this model [standard error [SE] and 95% confidence interval [CI] are reported]. Model calibration was assessed using the Hosmer-Lemeshow test.

Variables with *p* < 0.10 in univariable analysis were considered for inclusion in the multivariable model. A purposeful selection approach followed: variables that were non-significant and did not substantially influence the coefficients of key predictors were sequentially excluded. The final adjusted model included age over 35 years, obesity, history of spontaneous abortion, and preconception care. The AUC was calculated based on the predicted probabilities of this final model (outcome SA = 1).

Multicollinearity was assessed using variance inflation factors (VIF), and all predictors had acceptable VIF values (<2). The linearity of continuous variables was confirmed through scatterplot analysis. Odds ratios (OR) with 95% confidence intervals (CI) were used to report the results of the model.

## 3 Results

### 3.1 Socio-demographic and clinical characteristics of the original sample

Table 1 presents the socio-demographic and clinical characteristics of the sample. More than half of the sample, 775 women (50.8%), were aged 27–35 years, one-third, 476 women (31.2%), were aged 18–26 years, and 275 women (18%) were over 35 years old. Regarding ethnic composition, Kazakhs accounted for 732 women (48%), Russians for 595 women (39%), and other nationalities for 199 women (13%). A majority, 1,172 women (76.8%), lived in urban areas, while 354 women (23.2%) lived in rural areas.

**Table 1:** Socio-demographic and clinical characteristics of study participants.

Variables	Socio-demographic characteristics	All participants (*n* = 1,526)
Woman's age, years	18–26	476 (31.2%)
	27–35	775 (50.8%)
	>35	275 (18.0%)
Ethnicity	Kazakh	732 (48.0%)
	Russian	595 (39.0%)
	Others	199 (13.0%)
Residency	Urban	1,172 (76.8%)
	Rural	354 (23.2%)
Social status	Employed, civil servant	87 (5.7%)
	Employed, public sector worker	111 (7.3%)
	Employed, private sector worker	59 (3.9%)
	Employed, self-employed	27 (1.8%)
	Employed, laborer	9 (0.6%)
	Employed, other	112 (7.3%)
	Convicted	4 (0.3%)
	Other	683 (44.8%)
	Unemployed	23 (1.5%)
	Housewife	411 (26.9%)
Education	Secondary education	217 (14.2%)
	Higher than secondary education	1,309 (85.8%)
Marital status	Married	1,267 (83.0%)
	Not married	259 (17.0%)
Clinical characteristics		
BMI, kg/m^2^	Underweight	213 (14.0%)
	Normal weight	946 (62.0%)
	Overweight	274 (18.0%)
	Obese	93 (6.0%)
Type 1/ Type 2 diabetes	Yes	49 (3.2%)
	No	1,477 (96.8%)
Arterial hypertension	Yes	56 (3.7%)
	No	1,470 (96.3%)
Hypothyroidism	Yes	26 (1.7%)
	No	1,500 (98.3%)
Deficiency anemia	Yes	382 (25.0%)
	No	1,144 (75.0%)
Allergies	Yes	68 (4.5%)
	No	1,458 (95.5%)
Pre-pregnancy health insurance	Yes	1,329 (87.1%)
	No	197 (12.9%)
Parity	Primiparous	919 (60.2%)
	Multiparous	607 (39.8%)
History of SA	Yes	122 (8.0%)
	No	1,404 (92.0%)
Preconception care	Yes	930 (60.9%)
	No	596 (39.1%)

BMI, body mass index; SA, spontaneous abortion.

In terms of social status, 683 women (44.8%) were classified as “other,” 405 women (26.5%) were employed (including civil servants, state employees, employees of private organizations, and workers), 23 women (1.5%) were unemployed, and 411 women (26.9%) were housewives. At the time of the study, 217 women (14.2%) had secondary education, while 1,309 women (85.8%) had secondary specialized or higher education. Additionally, 1,267 women (83%) were married.

Among the women, 946 (62%) had a normal weight, 213 (14%) were underweight, 274 (18%) were overweight, and 93 (6%) suffered from first- or second-degree obesity. A history of diabetes mellitus type 1 or 2 was reported in 49 women (3.2%), arterial hypertension in 56 women (3.7%), hypothyroidism in 26 women (1.7%), and deficiency anemia in 382 women (25%). Additionally, 68 women (4.5%) suffered from allergies.

The majority, 1,329 women (87.1%), had medical insurance before pregnancy. This pregnancy was the first for 919 women (60.2%). A history of early pregnancy loss was reported in 122 women (8.0%). Moreover, 930 women (60.9%) had undergone preconception training.

### 3.2 Comparative groups of women with a favorable pregnancy outcome and first-trimester SA

The socio-demographic and clinical characteristics of women classified according to pregnancy outcome are presented in Table 2.

**Table 2:** Comparative groups of women with a favorable pregnancy outcome and first-trimester SA.

Variables		Favorable outcome (*n* = 1,236)	SA in the first trimester (*n* = 290)	*p*-value
Socio-demographic characteristics				
Age, years	18–26	399 (32.3%)	77 (26.6%)	**<0**.001*
	27–35	640 (51.8%)	135 (46.6%)	
	>35	197 (15.9%)	78 (26.9%)	
Ethnicity	Kazakh	593 (48.0%)	139 (47.9%)	0.725
	Russian	478 (38.7%)	117 (40.4%)	
	Other	165 (13.3)	34 (11.7%)	
Residency	Urban	941 (76.1%)	231 (79.7%)	0.201
	Rural	295 (23.9%)	59 (20.3%)	
Social status	Employed, civil servant	70 (5.7%)	17 (5.9%)	0.724
	Employed, public sector worker	92 (7.4%)	19 (6.6%)	
	Employed, private sector worker	48 (3.9%)	11 (3.8%)	
	Employed, self-employed	21 (1.7%)	6 (2.1%)	
	Employed, laborer	8 (0.6%)	1 (0.3%)	
	Employed, other	91 (7.4%)	21 (7.2%)	
	Convicted	4 (0.3%)	0 (0%)	
	Other	549 (44.4%)	134 (46.2%)	
	Unemployed	19 (1.5%)	4 (1.4%)	
	Housewife	334 (27.0%)	77 (26.6%)	
Education	Secondary education	177 (14.3%)	40 (13.8%)	0.818
	Higher than secondary education	1,059 (85.7%)	250 (86.2%)	
Marital status	Married	1,028 (83.2%)	239 (82.4%)	0.758
	Not married	208 (16.8%)	51 (17.6%)	
Clinical characteristics				
BMI, kg/m^2^	Underweight	168 (13.6%)	45 (15.5%)	**0**.053*
	Normal weight	777 (62.9%)	169 (58.2%)	
	Overweight	225 (18.2%)	49 (17.0%)	
	Obesity	66 (5.3%)	27 (9.3%)	
Type 1/Type 2 diabetes	Yes	34 (2.8%)	15 (5.2%)	**0**.036*
	Not	1,202 (97.2%)	275 (94.8%)	
Arterial hypertension	Yes	41 (3.3%)	15 (5.2%)	0.131
	No	1,195 (96.7%)	275 (94.8%)	
Hypothyroidism	Yes	21 (1.7%)	5 (1.7%)	0.977
	No	1,215 (98.3%)	285 (98.3%)	
Deficiency anemia	Yes	310 (25.1%)	72 (24.8%)	0.929
	No	926 (74.9%)	218 (75.2%)	
Allergies	Yes	51 (4.1%)	17 (5.9%)	0.198
	No	1,185 (95.9%)	273 (94.1)	
Pre-pregnancy health insurance	Yes	1,078 (87.2%)	251 (86.6%)	0.762
	No	158 (12.8%)	39 (13.4%)	
Parity	Primiparous	739 (59.8%)	180 (62.1%)	0.476
	Multiparous	497 (40.2%)	110 (37.9%)	
History of SA	Yes	90 (7.3%)	32 (11.0%)	**0**.034*
	No	1,146 (92.7%)	258 (89.0%)	
Preconception care	Yes	786 (63.6%)	144 (49.7%)	**<0**.001*
	Not	450 (36.4%)	146 (50.3%)	

BMI, body mass index; SA, spontaneous abortion.

* Bold values represent statistically significant results (*p* < 0.05).

In the group with first-trimester SA, compared to women with a favorable pregnancy outcome, there was a higher proportion of women over 35 years of age (26.9% vs. 15.9%). Additionally, women with SA were more likely to live in urban areas (79.7% vs. 76.1%), have higher than secondary education (86.2% vs. 85.7%), and be unmarried (17.6% vs. 16.8%).

Clinically, women in the first-trimester SA group had a higher prevalence of obesity (9.3% vs. 5.3%), type 1 or 2 diabetes (5.2% vs. 2.8%), arterial hypertension (5.2% vs. 3.3%), and allergies (5.9% vs. 4.1%). Furthermore, a larger proportion of these women had a history of SA (11% vs. 7.3%). In contrast, relatively fewer women in the SA group underwent preconception care compared to those with a favorable pregnancy outcome (49.7% vs. 63.6%).

The results of the univariate analysis of risk factors for SA in the study sample are presented in Table 3.

**Table 3:** Univariable and multivariable logistic regression for all candidate variables.

Factors	Favorable outcome (%) (*n* = 1,236)	SA in the first trimester (%) (*n* = 290)	Univariable analysis [OR (95% CI)]	*p*-value	Multivariable analysis [OR (95% CI)]	*p*-value
Age of the woman (years): >35	15.9 (197/1,236)	26.9 (78/290)	1.94 (1.44–2.62)	0.001	2.02 (1.49–2.75)	0.001
Ethnicity						
Kazakh	48.0 (593/1,236)	47.9 (139/290)	1.14 (0.75–1.72)	0.541	–	–
Russian	38.7 (478/1,236)	40.4 (117/290)	1.19 (0.78–1.81)	0.423		
Other	13.3 (165/1,236)	11.7 (34/290)	1.00	–		
Residency:						
Urban	76.1 (941/1,236)	79.7 (231/290)	1.23 (0.89–1.68)	0.201	–	–
Rural	23.9 (295/1,236)	20.3 (59/290)	1.00	–		
Social status						
Employed, civil servant	5.7 (70/1,236)	5.9 (17/290)	0.99 (0.57–1.75)	0.986	–	–
Employed, public sector worker	7.4 (92/1,236)	6.6 (19/290)	0.85 (0.50–1.44)	0.536		
Employed, private sector worker	3.9 (48/1,236)	3.8 (11/290)	0.94 (0.47–1.86)	0.856		
Employed, self-employed	1.7 (21/1,236)	2.1 (6/290)	1.17 (0.46–2.96)	0.739		
Employed, laborer	0.6 (8/1,236)	0.3 (1/290)	0.51 (0.06–4.13)	0.530		
Employed, other	7.4 (91/1,236)	7.2 (21/290)	0.95 (0.57–1.58)	0.830		
Convicted	0.3 (4/1,236)	0 (0/290)	0.45 (0.02–8.48)	0.597		
Other	44.4 (549/1,236)	46.2 (134/290)	1.00	–		
Unemployed	1.5 (19/1,236)	1.4 (4/290)	0.86 (0.29–2.58)	0.791		
Housewife	27.0 (334/1,236)	26.6 (77/290)	0.94 (0.69–1.29)	0.720		
Education	5.7 (70/1,236)	5.9 (17/290)	0.99 (0.57–1.75)	0.986		
Secondary education	14.3 (177/1,236)	13.8 (40/290)	0.96 (0.66–1.39)	0.817	–	–
Higher than secondary education	85.7 (1,059/1,236)	86.2 (250/290)	1.00	1.00		
Marital status						
Married	83.2 (1,028/1,236)	82.4 (239/1,236)	0.95 (0.68–1.33)	0.757	–	–
Not married	16.8 (208/1,236)	17.6 (51/1,236)	1.00			
BMI (kg/m^2^): Obesity	5.3 (66/1,236)	9.3 (27/290)	1.82 (1.14–2.90)	0.012	1.81 (1.12–2.91)	0.015
Type 1 or Type 2 Diabetes	2.8 (34/1,236)	5.22 (15/290)	1.93 (1.04–3.59)	0.038	–	–
Arterial Hypertension	3.3 (41/1,236)	5.22 (15/290)	1.59 (0.87–2.91)	0.134	–	–
Hypothyroidism	1.7 (21/1,236)	1.7 (5/290)	1.02 (0.38–2.72)	0.976	–	–
Deficiency anemia	25.1 (310/1,236)	24.8 (72/290)	0.99 (0.73–1.32)	0.929	–	–
Allergies	4.1 (51/1,236)	5.9 (17/290)	1.45 (0.82–2.54)	0.200	–	–
Pre-pregnancy health insurance	87.2 (1,078/1,236)	86.6 (251/290)	0.94 (0.65–1.37)	0.761	–	–
Parity						
Primiparous	59.8 (739/1,236)	62.1 (180/290)	1.10 (0.85–1.43)	0.475	–	–
Multiparous	40.2 (497/1,236)	37.9 (110/290)	1.00	–		
History of SA	7.3 (90/1,236)	11.0 (32/290)	1.58 (1.03–2.42)	0.035	1.57 (1.01–2.43)	0.043
Preconception Care	63.6 (786/1,236)	49.7 (144/290)	0.57 (0.44–0.73)	0.001	0.58 (0.45–0.75)	0.001

BMI, body mass index; SA, spontaneous abortion; OR, odds ratio; CI, confidence interval. The univariable analysis included all variables from Table 2. The multivariable analysis included only variables retained in the final adjusted model; “–” indicates that the variable was not included. The outcome is coded as SA = 1.

Univariate logistic regression analysis revealed that women aged over 35 years [[OR] = 1.94, 95% [CI] = 1.44–2.62], obesity [[OR] = 1.82, 95% [CI] = 1.14–2.90], type 1 or type 2 diabetes [[OR] = 1.93, 95% [CI] = 1.04–3.59], and a history of miscarriage [[OR] = 1.58, 95% [CI] = 1.03–2.42] were associated with an increased risk of first-trimester SA. Preconception care, however, was associated with a reduced risk of first-trimester SA [[OR] = 0.57, 95% [CI] = 0.44–0.73].

**Figure 2:**
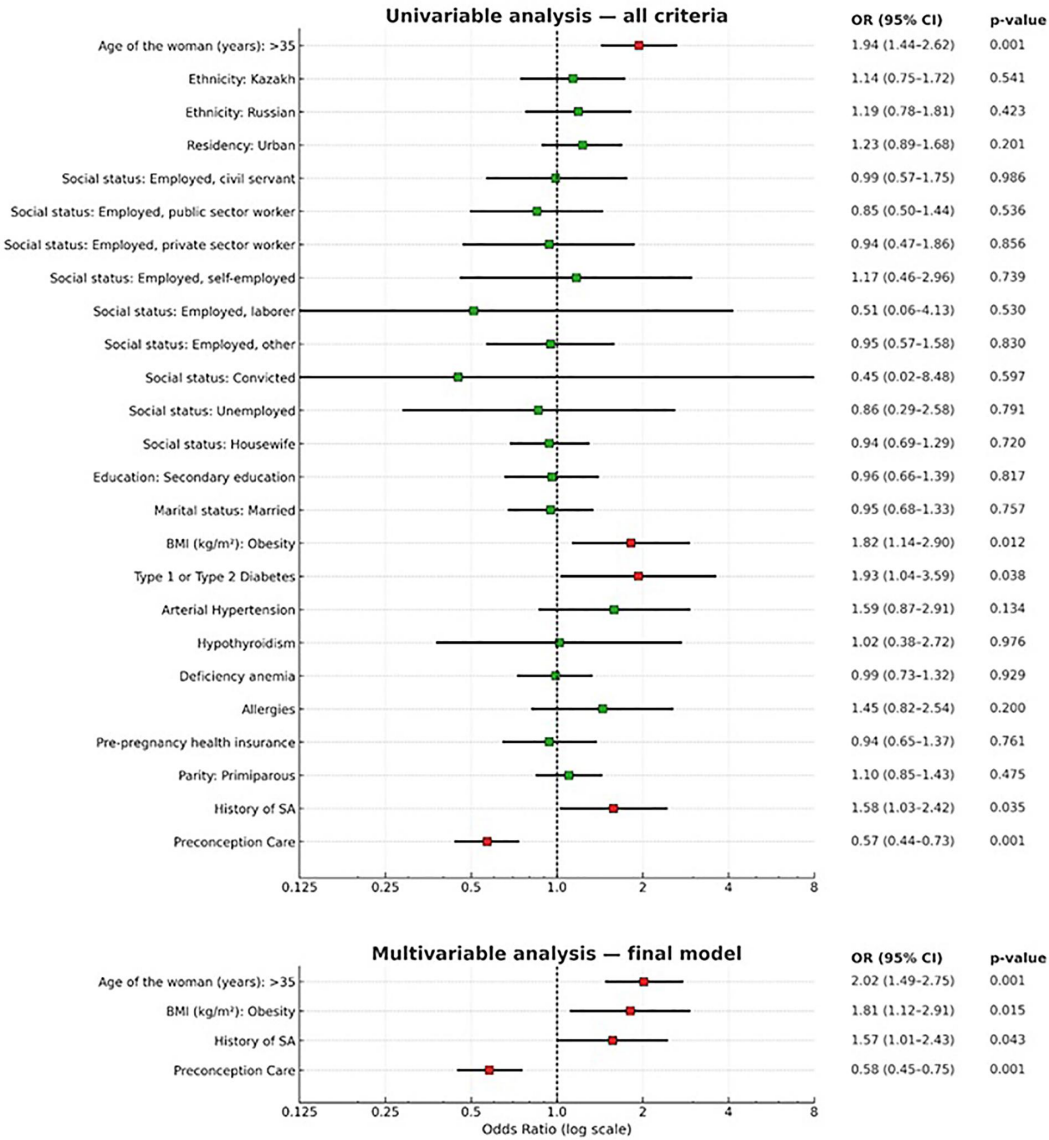
Risk factors for spontaneous abortion in the first trimester: univariate analysis and multivariate analysis (final adjusted model): age over 35 years, obesity, history of spontaneous abortion, and preconception care. Points represent odds ratios (OR) with 95% confidence intervals (CI); the vertical dashed line indicates OR=1 (no effect). The ROC-AUC, presented in the “Results” section, is calculated based on the predicted probabilities of the final adjusted model. The outcome is coded as SA=1.

Figure 2; Table 3 summarize the results of the multivariate logistic regression analysis. Independent factors influencing first-trimester SA included the woman's age, BMI, history of SA, and preconception care (*p* < 0.05). Women aged over 35 had an increased likelihood of first-trimester SA compared to those under 35 [[OR] = 2.02, 95% [CI] = 1.49–2.75]. Obese women were more likely to experience first-trimester SA than women with a normal BMI [[OR] = 1.81, 95% [CI] = 1.12–2.91]. The history of SA was also strongly associated with a higher risk of first-trimester SA [[OR] = 1.57, 95% [CI] = 1.01–2.43]. Women who received preconception care had a significantly reduced risk of first-trimester SA compared to those who did not [[OR] = 0.58, 95% [CI] = 0.45–0.75].

The final adjusted model included age over 35 years, obesity, history of spontaneous abortion, and preconception care. The discriminative ability of the model was moderate: AUC = 0.611 (SE 0.019; 95% CI 0.574–0.648; *p* < 0.001 compared with 0.50). Model calibration was satisfactory: Hosmer–Lemeshow test *χ*^2^ = 2.04, df = 4, *p* = 0.73.

## 4 Discussion

For practical healthcare, a key conceptual issue is the variability of risk factors for spontaneous miscarriage, as potentially modifiable factors can form the basis for clinical or preventive interventions in pregnancy management. However, given that most miscarriages occur in the early weeks of pregnancy, sometimes even before a woman knows she is pregnant, addressing these factors at this stage may be too late for any intervention or treatment to influence the outcome. This highlights the need to identify high-risk individuals before conception to implement appropriate preventive measures aimed at reducing the risk (32).

We assessed the socio-demographic and clinical risk factors for first-trimester spontaneous abortion. Our results showed that the odds of first-trimester SA were higher in women over 35 years of age compared to women under 35 [[OR] = 2.02, 95% [CI] = 1.49–2.75]. This finding is consistent with previous research (33–35). The decline in reproductive potential in older women may be attributed to reduced oocyte quality, changes in the endometrium, and altered progesterone levels (36). Although age is a non-modifiable risk factor at the individual level, especially in cases of unintended pregnancies, it can be considered a modifiable risk factor at the population level. Including this factor in risk assessments can help prevent underestimating the risk of miscarriage in this population.

Furthermore, a strong negative impact of high BMI on pregnancy outcomes was observed [[OR] = 1.81, 95% [CI] = 1.12–2.91]. The risk of SA among women with obesity in our study aligns with findings from previous studies (37, 38). Possible mechanisms include poor endometrial receptivity, a pro-inflammatory state caused by cytokines, hormonal imbalances, or impaired blood supply to the endometrium and placenta (39–41). Further research is needed to confirm these theories. Early weight loss interventions may reduce the risk of SA in obese patients. Public health policies targeting obesity prevention can significantly improve women's reproductive outcomes (42). Such interventions should adopt a multisectoral approach addressing social, structural, economic, and environmental factors. Raising awareness among women about the importance of lifestyle changes and modifiable risk factors is necessary for reducing the overall risk of SA and other adverse maternal and infant health outcomes (12, 43–45).

We also found that women with a history of SA had an increased risk of first-trimester SA [[OR] = 1.57, 95% [CI] = 1.01–2.43], consistent with prior research indicating a significantly higher risk in this group (33). While previous pregnancy loss is a non-modifiable factor, including it in risk assessments can prevent underestimating the risk in these populations.

An important finding of our study was that women who received preconception care had a lower risk of first-trimester SA [[OR] = 0.58, 95% [CI] = 0.45–0.75] compared to those who did not receive preconception care. This reinforces the conclusion that preconception care improves pregnancy outcomes (46). The preconception period offers a critical window for intervention, empowering women at higher risk of SA to address modifiable risk factors before conception (47). Potential mechanisms for reducing the risk of early pregnancy loss include optimizing chronic disease management, correcting nutritional deficiencies, normalizing weight, reducing harmful exposures, preventing infections, and taking folic acid, which aligns with meta-analysis findings (18, 48). These all highlight the need for national initiatives to improve the health of women of childbearing age before planned pregnancies. In Kazakhstan, the importance of preconception care has been recognized with the implementation of a new clinical protocol in 2,023, providing comprehensive guidelines for healthcare providers to ensure its effective delivery (49). This protocol emphasizes the screening for chronic diseases, nutritional deficiencies, lifestyle modifications, vaccination, psychosocial support, counseling, and education to prepare couples for the emotional and physical challenges of pregnancy and parenthood.

The primary strength of this study is that, to the best of our knowledge, it is the first to investigate preconception care as a factor influencing first-trimester SA in women in Kazakhstan. Similar studies have been conducted in high-income countries with advanced healthcare systems, such as the United States and the United Kingdom, focusing on risk factors for early pregnancy loss and the broader implications of miscarriage (50, 51). Our study, conducted in Kazakhstan, applies to other Central Asian countries due to similar healthcare systems and their classification as middle-income economies. The analysis included a large sample of 1,526 women, which strengthens the statistical power and reliability of the results. These findings guide clinical counseling on spontaneous abortion risks and the public health benefits of preconception care. The study shows that preconception care protects against early pregnancy loss, providing clear support for clinical practice and public health efforts to improve pregnancy outcomes. This makes the results relevant for shaping policies and allocating resources. Integrating preconception care into routine health services, especially in low- and middle-income countries with limited access to such interventions, addresses modifiable risk factors like obesity and advanced maternal age. Such efforts improve maternal and neonatal health, lower healthcare costs, and promote health equity.

In addition to the factors identified in our analysis, long-term changes in the reproductive system, including hormonal disturbances, may also influence pregnancy outcomes. Emerging data suggest that some of these changes may be associated with prior COVID-19 infection, potentially affecting menstrual cycle regularity, ovulatory function, and endometrial receptivity (52–54). Although the clinical significance of these observations requires further clarification, including such variables in future studies will enhance understanding of the mechanisms underlying adverse pregnancy outcomes and support the development of more precise preventive strategies.

From a public health perspective, the study has several limitations. First, selection bias is possible: the data were obtained from a single regional hospital with two perinatal centers that admit medium- and high-risk patients, and records with incomplete data were excluded. Second, information bias is possible: data and outcomes were extracted from an electronic medical database, and several covariates were missing, such as income, smoking, alcohol use, gestational age, antenatal complications, use of assisted reproductive technologies, or male factors (55, 56). Third, residual bias remains, since assignment to preconception care was not randomized, and exclusion of records with missing data may introduce selection bias if missingness is related to both exposure and outcome. The study period coincided with COVID-19 waves, which could have affected access to care, health-seeking behavior, and the structure of recorded cases. The moderate discriminative ability of the model (ROC-AUC ≈0.62) suggests that the results are most useful for assessing associations and planning prevention at the population level rather than for individual risk prediction. In addition, binary dichotomization of the outcome does not capture the severity of perinatal outcomes among live births and excludes late fetal or neonatal deaths. Future research should apply composite or ordinal perinatal endpoints. Finally, the reliance on retrospective data from medical records may underestimate the true prevalence of risk factors, particularly for socially sensitive or less commonly documented variables such as lifestyle behaviors or preconception care practices.

## 5 Conclusion

In our study, we evaluated associations between candidate factors and spontaneous abortion in the first trimester. Age >35 years, obesity, and a history of spontaneous abortion were associated with higher odds of early pregnancy loss, whereas preconception care was associated with lower odds. These findings expand the understanding of preventive measures taken before pregnancy in relation to adverse outcomes in the first trimester. In clinical practice, they may support risk stratification and counseling of women who could benefit from targeted preventive or therapeutic interventions before conception. From a public health perspective, the observed association with preconception care should be interpreted as associative rather than causal, and further prospective studies are needed before drawing conclusions about its impact on maternal and neonatal outcomes.

## Data Availability

The data analyzed in this study is subject to the following licenses/restrictions: data is unavailable due to ethical and legal restrictions. Requests to access these datasets should be directed to the Republican state enterprise on the right of economic management "Republican Center for Healthcare Development" of the Ministry of Health of the Republic of Kazakhstan, office@nrchd.kz

## References

[1] World Health Organization. International classification of diseases (ICD). ICD-10 Version. (2019). Available online at: https://icd.who.int/browse10/2019/en (Accessed January 17, 2024).

[2] Alves C Jenkins SM Rapp A. Early pregnancy loss (spontaneous abortion). StatPearls. Tampa/St. Petersburg, FL: StatPearls Publishing (2023). Available online at: https://www.ncbi.nlm.nih.gov/books/NBK560521/ (Accessed January 17, 2024). 32809356

[3] Committee on Practice Bulletins—Gynecology. The American college of obstetricians and gynecologists practice bulletin no. 150. Early pregnancy loss. Obstet Gynecol. (2015) 125:1258–67. 10.1097/01.AOG.0000465191.27155.25 25932865

[4] Strumpf E Lang A Austin N Derksen SA Bolton JM Brownell MD Prevalence and clinical, social, and health care predictors of miscarriage. BMC Pregnancy Childbirth. (2021) 21:1–9. 10.1186/S12884-021-03682-Z/TABLES/2 33673832 PMC7936485

[5] Hardy K Hardy PJ. 1st Trimester miscarriage: four decades of study. Transl Pediatr. (2015) 4:189. 10.3978/J.ISSN.2224-4336.2015.03.05 26835373 PMC4729087

[6] Cohain JS Buxbaum RE Mankuta D. Spontaneous first trimester miscarriage rates per woman among parous women with 1 or more pregnancies of 24 weeks or more. BMC Pregnancy Childbirth. (2017) 17:1–7. 10.1186/S12884-017-1620-1/TABLES/3 29272996 PMC5741961

[7] Blushinova AN Shalgumbayeva GM Rakhmetova VS Ryspaeva ZA Aldabekova GU. Abortion practices among women in Kazakhstan: cross-sectional study. Nauka I Zdravookhranenie (Science & Healthcare). (2024) 26(2):76–81. 10.34689/SH.2024.26.2.010

[8] Sakko Y Turesheva A Gaipov A Aimagambetova G Ukybassova T Marat A Epidemiology of spontaneous pregnancy loss in Kazakhstan: a national population-based cohort analysis during 2014–2019 using the national electronic healthcare system. Acta Obstet Gynecol Scand. (2023) 102:1682. 10.1111/AOGS.14669 37667510 PMC10619606

[9] Pendina AA Krapivin MI Chiryaeva OG Petrova LI Pashkova EP Golubeva A V Chromosomal abnormalities in miscarriages and maternal age: new insights from the study of 7118 cases. Cells. (2024) 14:8. 10.3390/CELLS14010008 39791709 PMC11720377

[10] Kim YJ Lee JE Kim SH Shim SS Cha DH. Maternal age-specific rates of fetal chromosomal abnormalities in Korean pregnant women of advanced maternal age. Obstet Gynecol Sci. (2013) 56:160. 10.5468/OGS.2013.56.3.160 24327996 PMC3784117

[11] Zhanseitova Advisor A Crape B Astana MB. Spontaneous Abortion in Astana: Associated Factors, 2015–2017. Astana: Nazarbayev University School of Medicine (2018). Available online at: http://nur.nu.edu.kz/handle/123456789/3314 (Accessed January 17, 2024).

[12] Metwally M Ong KJ Ledger WL Li TC. Does high body mass index increase the risk of miscarriage after spontaneous and assisted conception? A meta-analysis of the evidence. Fertil Steril. (2008) 90:714–26. 10.1016/J.FERTNSTERT.2007.07.1290 18068166

[13] Muhammad T Wan Y Lv Y Li H Naushad W Chan WY Maternal obesity: a potential disruptor of female fertility and current interventions to reduce associated risks. Obes Rev. (2023) 24:e13603. 10.1111/OBR.13603 37452501

[14] Syböck K Hartmann B Kirchengast S. Maternal prepregnancy obesity affects foetal growth, birth outcome, mode of delivery, and miscarriage rate in Austrian women. Int J Environ Res Public Health. (2023) 20:4139. 10.3390/IJERPH20054139 36901147 PMC10002339

[15] Huan Z Yongping L Lu L Min Z Xingzhi C Yulong Q Maternal pre-pregnancy risk factors for miscarriage from a prevention perspective: a cohort study in China. Eur J Obstet Gynecol. (2016) 206:57–63. 10.1016/j.ejogrb.2016.07.514 27639132

[16] Yang J Wang Y Wang XY Zhao YY Wang J Zhao YY. Adverse pregnancy outcomes of patients with history of first-trimester recurrent spontaneous abortion. Biomed Res Int. (2017) 2017:4359424. 10.1155/2017/4359424 28798930 PMC5536133

[17] Sun H Mao J Su X Du Q. Impact of spontaneous abortion history and induced abortion history on perinatal outcomes of singleton pregnancies. BMC Public Health. (2023) 23:1–10. 10.1186/S12889-023-17264-5/TABLES/5 38031055 PMC10685546

[18] Magnus MC Hockey RL Håberg SE Mishra GD. Pre-pregnancy lifestyle characteristics and risk of miscarriage: the Australian longitudinal study on women’s health. BMC Pregnancy Childbirth. (2022) 22:1–10. 10.1186/S12884-022-04482-9/TABLES/3 35232386 PMC8887017

[19] Al-Alami Z Abu-Huwaij R Hamadneh S Taybeh E. Understanding miscarriage prevalence and risk factors: insights from women in Jordan. Medicina (B Aires). (2024) 60:1044. 10.3390/MEDICINA60071044 PMC11279235 39064473

[20] Feodor Nilsson S Andersen PK Strandberg-Larsen K Nybo Andersen AM. Risk factors for miscarriage from a prevention perspective: a nationwide follow-up study. BJOG. (2014) 121:1375–85. 10.1111/1471-0528.12694 24548778

[21] Preconception Care. Geneva: World Health Organization (WHO) (2017). Available online at: https://iris.who.int/handle/10665/205637 (Accessed January 18, 2024).

[22] Xu J Li X Zhou Q. Nationwide-free preconception care strategy: experience from China. Front Public Health. (2022) 10:934983. 10.3389/FPUBH.2022.934983/BIBTEX 36339191 PMC9626826

[23] Dean S V Lassi ZS Imam AM Bhutta ZA. Preconception care: closing the gap in the continuum of care to accelerate improvements in maternal, newborn and child health. Reprod Health. (2014) 11:1–8. 10.1186/1742-4755-11-S3-S1/FIGURES/3 25414942 PMC4196556

[24] Poix S Elmusharaf K. Investigating the pathways from preconception care to preventing maternal, perinatal and child mortality: a scoping review and causal loop diagram. Prev Med Rep. (2023) 34:102274. 10.1016/J.PMEDR.2023.102274 37387730 PMC10302151

[25] Clinical Protocol for the Diagnosis and Treatment of Pregnancy Loss Approved by the Unified Commission for the Quality of Medical Services of the Ministry of Health of the Republic of Kazakhstan on July 28, 2023, Protocol No. 185. Available online at: https://nrchd.kz/ru/2017-03-12-10-51-13/klinicheskie-protokoly (Accessed January 18, 2024).

[26] Early Pregnancy Loss. Washington, DC: American College of Obstetricians and Gynecologists (ACOG) (2018). Available online at: https://www.acog.org/clinical/clinical-guidance/practice-bulletin/articles/2018/11/early-pregnancy-loss (Accessed January 18, 2024).

[27] On Approval of the Standard for Organizing Obstetric and Gynecological Care in the Republic of Kazakhstan Order of the Minister of Health of the Republic of Kazakhstan No-92, August 26, 2021. Available online at: https://adilet.zan.kz/kaz/docs/V2100024131 (Accessed January 18, 2024).

[28] Obesity: Preventing and Managing the Global Epidemic: Report of a WHO Consultation. Geneva: World Health Organization (WHO) (2000). Available online at: https://iris.who.int/handle/10665/42330 (Accessed January 18, 2024). 11234459

[29] Choo V. WHO Reassesses appropriate body-mass index for Asian populations. Lancet. (2002) 360:235. 10.1016/S0140-6736(02)09512-0 12133671

[30] Clinical Protocol for the Diagnosis and Treatment of Arterial Hypertension Approved by the Unified Commission for the Quality of Medical Services of the Ministry of Health of the Republic of Kazakhstan on October 3, 2019, Protocol No. 74. Available online at: https://online.zakon.kz/Document/?doc_id=37136070&pos=3;-44#pos=3;-44 (Accessed January 18, 2024).

[31] World Health Organization. WHO Recommendations on antenatal care for a positive pregnancy experience. WHO Recommendations on Antenatal Care for a Positive Pregnancy Experience. Geneva: World Health Organization (WHO) (2016). Available online at: https://www.ncbi.nlm.nih.gov/books/NBK409108/ (Accessed January 18, 2024).

[32] Taizhanova D Zubkov DV Komlichenko EV Magalov I Sorokina MA Bespalova NV The possibilities of adverse pregnancy outcomes predicting based on laboratory markers of reproductive losses. Med Ecol. (2025) 0:77–84. 10.59598/ME-2305-6045-2024-113-4-77-84

[33] Magnus MC Wilcox AJ Morken NH Weinberg CR Håberg SE. Role of maternal age and pregnancy history in risk of miscarriage: prospective register based study. Br Med J. (2019) 364:869. 10.1136/BMJ.L869 PMC6425455 30894356

[34] Cavazos-Rehg PA Krauss MJ Spitznagel EL Bommarito K Madden T Olsen MA Maternal age and risk of labor and delivery complications. Matern Child Health J. (2015) 19:1202–11. 10.1007/S10995-014-1624-7 25366100 PMC4418963

[35] Koo YJ Ryu HM Yang JH Lim JH Lee JE Kim MY Pregnancy outcomes according to increasing maternal age. Taiwan J Obstet Gynecol. (2012) 51:60–5. 10.1016/J.TJOG.2012.01.012 22482970

[36] Soler A Morales C Mademont-Soler I Margarit E Borrell A Borobio V Overview of chromosome abnormalities in first trimester miscarriages: a series of 1,011 consecutive chorionic villi sample karyotypes. Cytogenet Genome Res. (2017) 152:81–9. 10.1159/000477707 28662500

[37] Ghimire PR Akombi-Inyang BJ Tannous C Agho KE. Association between obesity and miscarriage among women of reproductive age in Nepal. PLoS One. (2020) 15:e0236435. 10.1371/JOURNAL.PONE.0236435 32760090 PMC7410243

[38] Silvestris E de Pergola G Rosania R Loverro G. Obesity as disruptor of the female fertility. Reprod Biol Endocrinol. (2018) 16:1–13. 10.1186/S12958-018-0336-Z 29523133 PMC5845358

[39] Snider AP Wood JR. Obesity induces ovarian inflammation and reduces oocyte quality. Reproduction. (2019) 158:R79–90. 10.1530/REP-18-0583 30999278

[40] Metwally M Preece R Thomas J Ledger W Li TC. A proteomic analysis of the endometrium in obese and overweight women with recurrent miscarriage: preliminary evidence for an endometrial defect. Reprod Biol Endocrinol. (2014) 12:75. 10.1186/1477-7827-12-75 25096020 PMC4237937

[41] Macroscopic and Microscopic Morphological Features of The Placenta Associated with Preeclampsia and Chronic Hypoxic Damage of the Fetus | Muhammad | Medicine and ecology. (Accessed January 22, 2024).

[42] Kalantari E Tajvar M Naderimagham S Takian A. Maternal obesity management: a narrative literature review of health policies. BMC Womens Health. (2024) 24:1–12. 10.1186/S12905-024-03342-2/TABLES/4 39294652 PMC11409689

[43] Belan M Harnois-Leblanc S Laferrère B Baillargeon JP. Optimizing reproductive health in women with obesity and infertility. CMAJ. (2018) 190:E742. 10.1503/CMAJ.171233 29914911 PMC6008192

[44] Nukeshtayeva K Kayupova G Yerdessov N Bolatova Z Zhamantayev O Turmukhambetova A. Factors associated with maternal mortality in Kazakhstan: a pre- and during-pandemic comparison. Front Public Health. (2024) 12:1337564. 10.3389/FPUBH.2024.1337564 38887251 PMC11180802

[45] Yerdessov N Zhamantayev O Bolatova Z Nukeshtayeva K Kayupova G Turmukhambetova A. Infant mortality trends and determinants in Kazakhstan. Children. (2023) 10:923. 10.3390/CHILDREN10060923 37371155 PMC10297167

[46] Timraz M Abu-Hamad B Ibaid A. Does preconception care make a difference to pregnancy outcomes? A quasi-experimental, mixed-methods study. Lancet. (2022) 399:S44. 10.1016/s0140-6736(22)01179-5

[47] Benedetto C Borella F Divakar H O’Riordan SL Mazzoli M Hanson M FIGO preconception checklist: preconception care for mother and baby. Int J Gynaecol Obstet. (2024) 165:1–8. 10.1002/IJGO.15446 38426290

[48] Wahabi HA Fayed A Esmaeil S Elmorshedy H Titi MA Amer YS Systematic review and meta-analysis of the effectiveness of pre-pregnancy care for women with diabetes for improving maternal and perinatal outcomes. PLoS One. (2020) 15(8):e0237571. 10.1371/journal.pone.0237571 32810195 PMC7433888

[49] Clinical Protocol for Diagnosis and Treatment: Preconception Care, approved by the Join Kat Commission on the Quality of Medical Services of the Ministry of Health of the Republic of Kazakhstan, dated July 28, 2023, Protocol No. 185. Available online at: https://nrchd.kz/ru/2017-03-12-10-51-13/klinicheskie-protokoly (Accessed January 20, 2024).

[50] Gracia CR Sammel MD Chittams J Hummel AC Shaunik A Barnhart KT. Risk factors for spontaneous abortion in early symptomatic first-trimester pregnancies. Obstet Gynecol. (2005) 106:993–9. 10.1097/01.AOG.0000183604.09922.E0 16260517

[51] Quenby S Gallos ID Dhillon-Smith RK Podesek M Stephenson MD Fisher J Miscarriage matters: the epidemiological, physical, psychological, and economic costs of early pregnancy loss. Lancet. (2021) 397:1658–67. 10.1016/S0140-6736(21)00682-6/ATTACHMENT/CD1B82A0-EB07-4548-B733-4399095F3D6E/MMC1.PDF 33915094

[52] Lebar V Laganà AS Chiantera V Kunič T Lukanović D. The effect of COVID-19 on the menstrual cycle: a systematic review. J Clin Med. (2022) 11:3800. 10.3390/jcm11133800 35807090 PMC9267255

[53] Ghaemi M Hantoushzadeh S Shafiee A Gargari OK Fathi H Eshraghi N The effect of COVID-19 and COVID-19 vaccination on serum anti-mullerian hormone: a systematic review and meta-analysis. Immun Inflamm Dis. (2024) 12:e1136. 10.1002/iid3.1136 38270314 PMC10777886

[54] Huang J Liu Y Wang J Xu D Huang Z Li M Pregnancy outcomes after frozen-thawed embryo transfer in women with COVID-19 history: a prospective cohort study. J Med Virol. (2024) 96:e29377. 10.1002/jmv.29377 38235921

[55] Kleinhaus K Perrin M Friedlander Y Paltiel O Malaspina D Harlap S. Paternal age and spontaneous abortion. Obstet Gynecol. (2006) 108:369–77. 10.1097/01.AOG.0000224606.26514.3A 16880308

[56] Robinson L Gallos ID Conner SJ Rajkhowa M Miller D Lewis S The effect of sperm DNA fragmentation on miscarriage rates: a systematic review and meta-analysis. Hum Reprod. (2012) 27:2908–17. 10.1093/HUMREP/DES261 22791753

