# Supplementary material for: Risk factors for first-trimester spontaneous abortion and the role of preconception care
Source: Front Glob Womens Health. 2025 Sep 23;6:1615983. doi: 10.3389/fgwh.2025.1615983 (PMC12500644; doi:10.3389/fgwh.2025.1615983)
Supplement: Supplementary file 1 [file Table1.docx]

Supplementary table 1. Socio-demographic and clinical characteristics of study participants (included and excluded groups)

| Criteria |  | Included  (n-1526)  n (%) | Excluded  (n-351)  n (%) | p-value |
| --- | --- | --- | --- | --- |
| *Socio-demographic characteristics* | | |  |  |
| **Woman's age, years** | 18-26 | 476 (31.2%) | 119 (33.9) | 0.572 |
|  | 27-35 | 775 (50.8%) | 180 (51.3) |  |
|  | > 35 | 275 (18.0%) | 52 (14.8) |  |
| **Ethnicity** | Kazakh | 732 (48.0%) | 161 (45.9) | 0.105 |
|  | Russian | 595 (39.0%) | 129 (36.8) |  |
|  | Others | 199 (13.0%) | 61 (17.4) |  |
|  | No data | - | - |  |
| **Residency** | Urban | 1172 (76.8) | 254 (72.4) | 0.08 |
|  | Rural | 354 (23.2) | 97 (27.6) |  |
|  | No data | - | - |  |
| **Social status** | Employed, civil servant | 87 (5.7) | 19 (5.4) | >0.05 |
|  | Employed, public sector worker | 111 (7.3) | 29 (8.3) |  |
|  | Employed, private sector worker | 59 (3.9) | 12 (3.4) |  |
|  | Employed, self-employed | 27 (1.8) | 6 (1.7) |  |
|  | Employed, laborer | 9 (0.6) | 3 (0.9) |  |
|  | Employed, other | 112 (7.3) | 11 (3.1) |  |
|  | Convicted | 4 (0.3) | 1 (0.3) |  |
|  | Other | 683 (44.8) | 22 (6.3) |  |
|  | Unemployed | 23 (1.5) | 7 (2.0) |  |
|  | Housewife | 411 (26.9) | 87 (24.8) |  |
|  | No data | - | 154 (43.9) |  |
| **Education** | Secondary education | 217 (14.2) | 45 (12.8) | <0.01 |
|  | Higher than secondary education | 1309 (85.8) | 235 (67.0) |  |
|  | No data | - | 71 (20.2) |  |
| **Marital status** | Married | 1267 (83.0) | 276 (78.6) | 0.053 |
|  | Not married | 259 (17.0) | 75 (21.4) |  |
|  | No data | - | - |  |
| *Clinical characteristics* | | |  |  |
| **BMI, kg /m2** | Underweight | 213 (14.0) | 45 (12.8) | 0.597 |
|  | Normal weight | 946 (62.0) | 211 (60.1) |  |
|  | Overweight | 274 (18.0) | 68 (19.4) |  |
|  | Obese | 93 (6.0) | 27 (7.7) |  |
| **Type 1/ Type 2 diabetes** | Yes | 49 (3.2) | 9 (2.6) | 0.528 |
|  | No | 1477 (96.8) | 342 (97.4) |  |
|  | No data | - | - |  |
| **Arterial hypertension** | Yes | 56 (3.7) | 19 (5.4) |  |
|  | No | 1470 (96.3) | 332 (94.6) |  |
|  | No data | - | - |  |
| **Hypothyroidism** | Yes | 26 (1.7) | 2 (0.6) | 0.115 |
|  | No | 1500 (98.3) | 349 (99.4) |  |
|  | No data | - | - |  |
| **Deficiency anemia** | Yes | 382 (25.0) | 75 (21.4) | 0.150 |
|  | No | 1144 (75.0) | 276 (78.6) |  |
|  | No data | - | - |  |
| **Allergies** | Yes | 68 (4.5) | 24 (6.8) | 0.063 |
|  | No | 1458 (95.5) | 327 (93.2) |  |
|  | No data | - | - |  |
| **Pre-pregnancy health insurance** | Yes | 1329 (87.1) | 314 (89.5) | 0.226 |
|  | No | 197 (12.9) | 37 (10.5) |  |
|  | No data | - | - |  |
| **Parity** | Primiparous | 919 (60.2) | 198 (56.4) | 0.190 |
|  | Multiparous | 607 (39.8) | 153 (43.6) |  |
|  | No data | - | - |  |
| **History of SA** | Yes | 122 (8.0) | 35 (10.0) | 0.106 |
|  | No | 1404 (92.0) | 316 (90.0) |  |
|  | No data | - | - |  |
| **Preconception care** | Yes | 930 (60.9) | 135 (38.4) | <0.01 |
|  | No | 596 (39.1) | 75 (21.4) |  |
|  | No data | - | 141 (40.2) |  |
